# Hepcidin detects iron deficiency in Sri Lankan adolescents with a high burden of hemoglobinopathy: A diagnostic test accuracy study

**DOI:** 10.1002/ajh.24617

**Published:** 2017-01-17

**Authors:** Katherine Wray, Angela Allen, Emma Evans, Chris Fisher, Anuja Premawardhena, Lakshman Perera, Rexan Rodrigo, Gayan Goonathilaka, Lebbe Ramees, Craig Webster, Andrew E Armitage, Andrew M Prentice, David J Weatherall, Hal Drakesmith, Sant‐Rayn Pasricha

**Affiliations:** ^1^ MRC Human Immunology Unit, MRC Weatherall Institute of Molecular Medicine, University of OxfordOxfordUK; ^2^ BRC Blood ThemeNIHR Oxford Biomedical Research CentreOxfordUK; ^3^ Liverpool School of Tropical MedicineCentre for Tropical and Infectious DiseasesLiverpoolUK; ^4^ Department of Biochemistry and ImmunologyBirmingham Heartlands HospitalBirminghamUK; ^5^ MRC Molecular Haematology Unit, MRC Weatherall Institute of Molecular Medicine, University of OxfordOxfordUK; ^6^ Department of MedicineUniversity of KelaniyaColomboSri Lanka; ^7^ MRC Unit The Gambia, MRC KenebaThe Gambia; ^8^ MRC International Nutrition Group, London School of Hygiene and Tropical MedicineLondonUK

## Abstract

Anemia affects over 800 million women and children globally. Measurement of hepcidin as an index of iron status shows promise, but its diagnostic performance where hemoglobinopathies are prevalent is unclear. We evaluated the performance of hepcidin as a diagnostic test of iron deficiency in adolescents across Sri Lanka.

We selected 2273 samples from a nationally representative cross‐sectional study of 7526 secondary schoolchildren across Sri Lanka and analyzed associations between hepcidin and participant characteristics, iron indices, inflammatory markers, and hemoglobinopathy states. We evaluated the diagnostic accuracy of hepcidin as a test for iron deficiency with estimation of the AUC^ROC^, sensitivity/specificity at each hepcidin cutoff, and calculation of the Youden Index to find the optimal threshold.

Hepcidin was associated with ferritin, sTfR, and hemoglobin. The AUC^ROC^ for hepcidin as a test of iron deficiency was 0.78; hepcidin outperformed Hb and sTfR. The Youden index‐predicted cutoff to detect iron deficiency (3.2 ng/mL) was similar to thresholds previously identified to predict iron utilization and identify deficiency in African populations. Neither age, sex, nor α‐ or β‐thalassemia trait affected diagnostic properties of hepcidin. Hepcidin pre‐screening would prevent most iron‐replete thalassemia carriers from receiving iron whilst still ensuring most iron deficient children were supplemented. Our data indicate that the physiological relationship between hepcidin and iron status transcends specific populations. Measurement of hepcidin in individuals or populations could establish the need for iron interventions.

## 1 Introduction

About 800 million women and children are anemic worldwide, with the prevalence highest in Asia and sub‐Saharan Africa.^1^ Reducing the burden of anemia is a core 2025 global nutrition target. These goals form the basis for the rationale for programmes aimed at improving iron stores where the burden of anemia is high. For example, India, where the prevalence of anemia in adolescents is 56–90%,^2^ offers weekly iron supplementation to over 100 million adolescent boys and girls.^3^

Elsewhere, anemia may be less prevalent, and importantly, may be attributable to causes other than iron deficiency. For example, in Cambodia only a minority of anemia in schoolchildren is attributable to iron deficiency, with hemoglobinopathy being the dominant cause.^4^ In Sri Lanka, the prevalence of anemia in adolescents is about 20%^2^; and hence this group may be a candidate for long term weekly iron supplementation. However, Sri Lanka also has a high concomitant burden of genetic hemoglobinopathy, including carriage of α‐globin deletions (especially α‐3.7), and ß‐globin mutations (ß‐thalassemia and Hemoglobin‐E).^5^ Importantly, there are established concerns regarding safety of universal iron programs due to risks of malaria^6^ and alterations in intestinal microbiota,^7^ and emerging concerns about risk of iron overload especially in populations at high risk of hemoglobinopathy. In settings such as Sri Lanka, universal iron may only benefit the minority with iron deficiency anemia, provide no benefit, and perhaps harm. Stratification of iron interventions may therefore be of value.

Hepcidin is the peptide hormone that regulates iron homeostasis.^8^ Plasma hepcidin directly mediates iron absorption and utilization from the intestine, and is upregulated by iron stores and inflammation and suppressed by iron deficiency and increased erythropoiesis.^9^ Hepcidin might be marginally suppressed in carriers of thalassemia,^10^ resulting in increased iron absorption in this group and perhaps exposing them to risks of iron overload when subjected to prolonged iron supplementation.^11^ Hepcidin has been evaluated as a potential indicator of iron status and iron absorption,^12^ and has been shown to select African children whom might benefit from iron supplementation.^13^ Hepcidin could provide a useful tool to direct iron to individuals in settings where, while anemia still presents an important public health problem, the prevalence may not warrant universal supplementation, or where other factors (e.g., thalassemia carriage) contribute greatly to anemia.

In a nationally representative study of Sri Lankan secondary school children we collected data from a sample enriched for individuals at risk of iron deficiency or thalassemia carriage. We investigated the relationship between hepcidin and iron status, with interactions for thalassemia, then evaluated the properties of hepcidin as a diagnostic test of iron deficiency in this population and identified the optimal diagnostic threshold for hepcidin.

## 2 Subjects and methods

### 2.1 Study design

We obtained samples from a nationally representative cross‐sectional study designed to assess the frequency and distribution of hemoglobin variants across Sri Lanka. From each of 25 districts we purposefully selected approximately three schools (72 in total, including 5 temporary schools for displaced students from the North) that were geographically spaced and representative of the population.^14^ Participants were eligible if they were attending a selected secondary school and were aged 12–19 years inclusive. Samples were collected between June 2009 and July 2010.

### 2.2 Laboratory measurements

We collected a 5 mL blood sample from each student and transferred it into EDTA and plain tubes. We measured Hb and red cell indices (Coulter Counter, Beckman Coulter UK) and identified Hb variants by High Performance Liquid Chromatography using the ß‐thalassemia short program (BioRad, India). DNA was tested for α‐globin gene deletions (3.7 and 4.2) by multiplex PCR.^15^ We measured serum ferritin and transferrin receptor (sTfR) by ELISA (IBL International and R&D systems, respectively), and inflammation (hsCRP, Architect C8000, Abbott Laboratories). The sTfR‐ferritin (sTfR‐F) index was calculated as sTfR/log_10_(ferritin). Results for diagnostic reference standards were not viewed by the operator measuring hepcidin.

We quantified serum hepcidin by competitive ELISA (hepcidin‐25 human bioactive ELISA and hepcidin‐25 High Sensitivity ELISA, DRG). The high sensitivity ELISA was introduced by DRG during the course of the study; we calculated an adjustment factor to ensure comparability based on data provided by DRG. We validated the adjustment by ensuring mean hepcidin concentrations were not different between control groups assayed using both kits, in which mean ferritin, sTfR, and CRP were also similar. The lower limit of detection (LOD), estimated as being the hepcidin value corresponding to 3SDs below the mean 0 ng/mL hepcidin standard OD450, was calculated to be 0.78 ng/mL. Samples that gave readings less than the LOD were reported as LOD/2 (0.39 ng/mL). Inter‐plate coefficients of variation (CV%) for the mean OD450 nm on the “old” DRG kit: high and low concentration controls (provided by kit) were 13.8 and 12.2, respectively (*n* = 56). Inter‐plate CV% for the mean OD450 nm on the new HS‐DRG kit for high and low controls (included with the assay) were 9.0% and 10.6%, respectively (*n* = 9).

Multiple commercial assays are available for hepcidin measurement and, whilst correlation has been shown to be good between some specific assays,^16^ each assay measures different absolute values of hepcidin, thereby preventing direct comparison of results between measurements obtained using different assays. For this reason, twenty‐eight additional samples were also run in parallel using the DRG hepcidin‐25 bioactive ELISA and the Bachem Hepcidin‐25 (human) Enzyme Immunoassay (run as previously described)^13^ to allow comparison of results between methods.

### 2.3 Statistics

Data were analyzed using Stata 11 (StataCorp., College Station, TX). Hepcidin, ferritin, sTfR, CRP, and sTfR‐F index data were skewed and variables were log_10_ transformed for analysis. Arithmetic or geometric means were calculated where appropriate along with the 2.5th to 97.5th centile range.

Using linear regression, we estimated associations between hepcidin, participant characteristics, inflammatory markers and iron indices. Statistical significance was defined as *P* < 0.05 and β coefficients were estimated by analysis of variables standardized such that their variances were 1. We performed stepwise multiple regression, and retained only those variables with *P* < 0.05. Regression diagnostics were performed and the optimal model selected. Missing data were handled by listwise deletion for particular analyses.

We used a gold standard definition of iron deficiency, termed “combined definition”, previously deployed in sub‐Saharan African children: serum ferritin < 15 ng/mL, or <30 ng/mL in the presence of CRP > 5 mg/L, and sTfR/log_10_ferritin (sTfR‐F) index > 2.^13^ This definition combines the current WHO definition of iron deficiency using ferritin (adjusted for inflammation),^17^ along with cutoffs of the sTfR‐F index.^18^ This “combined definition” ensures that individuals with ferritin concentrations between 15 and 30 ng/mL and inflammation are only considered iron deficient if they also demonstrate tissue iron depletion.^13^ In order to permit comparison of sTfR with hepcidin, we used a second gold standard: “ferritin alone,” reflecting current WHO guidelines: ferritin < 15 or <30 ng/mL in the presence of inflammation (CRP > 5 mg/L).^17^

Receiver operating characteristic (ROC) curves were graphed and area under the curve (AUC^ROC^) values calculated. We compared the AUC^ROC^ of hepcidin to detect iron deficiency between individuals with/without carriage of hemoglobinopathy. Sensitivities and specificities were calculated for each cutoff of hepcidin and the Youden index [(sensitivity + specificity) – 1] estimated. The ROC curves and Youden index were inspected to determine the point at which the sensitivity and specificity were both maximized.

### 2.4 Ethics

Approval for this research program was obtained from the Ethical Committee of the University of Kelaniya, Colombo, Sri Lanka; and the Oxford Tropical Research Ethical Committee, Oxford, United Kingdom. Permission for enrolment into the study was obtained from each of the schools and families involved.

### 2.5 Role of the funding source

The funding source had no role in the collection, analysis, and interpretation of data; in the writing of the report; or in the decision to submit the paper for publication. The corresponding authors had full access to all the data in the study and had final responsibility for the decision to submit for publication.

## 3 Results

**Figure 1:**
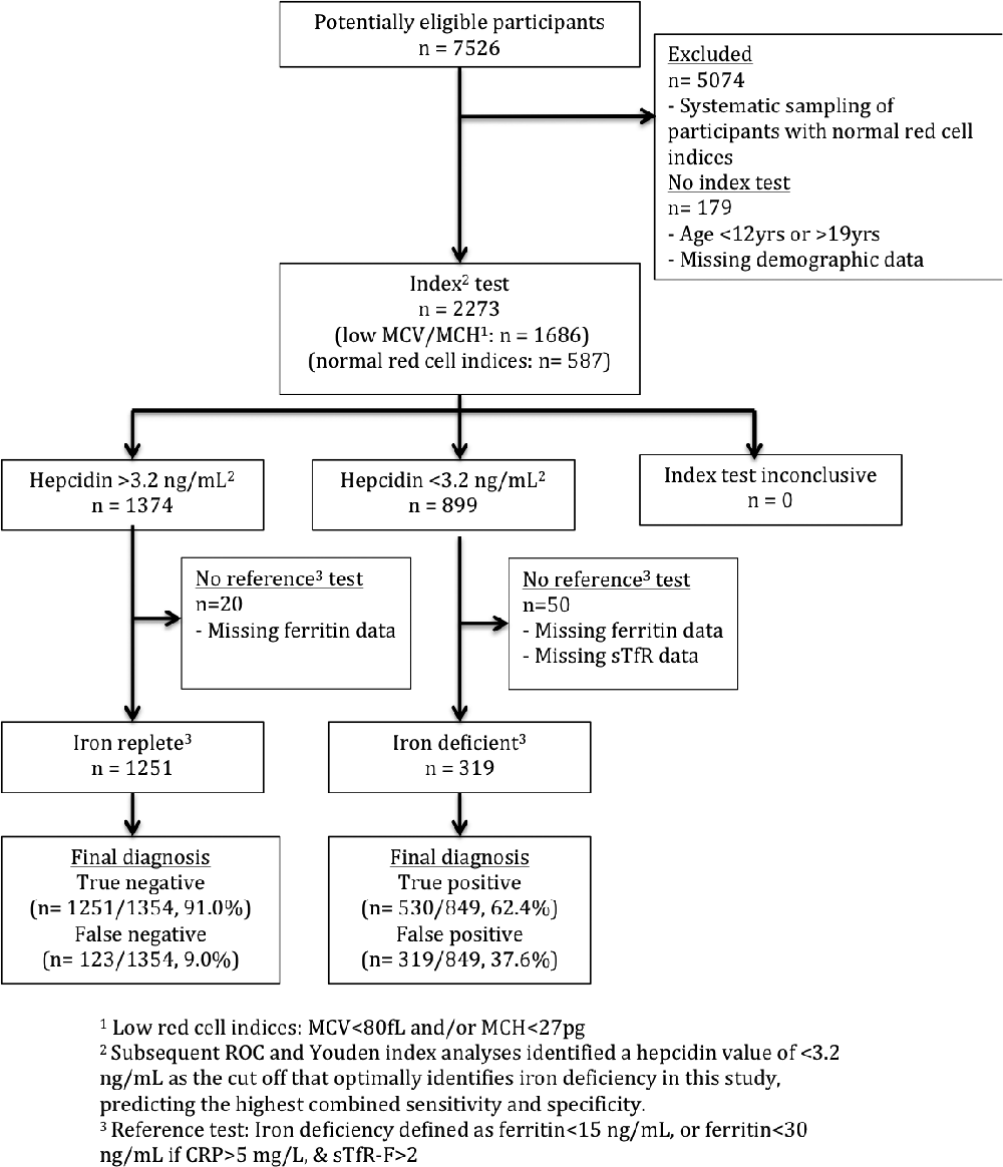
Flow of participants through the study

From a total of 7526 students we analyzed samples from 2273 children aged 12–19 years: 587 with normal red cell indices and 1686 with low red cell indices (MCV < 80 fL and/or MCH < 27 pg), enriching our dataset for hemoglobinopathies. The flow of participants is detailed in Figure 1.

Participant demographics and iron indices are summarized in Table 1. The prevalence of anemia within this selected study group was 19.8%, and using the combined definition iron deficiency was seen in 19.2% and was more prevalent in girls (27.6%) than boys (10.4%) (*P* < 0.001). Only 3.5% of children had inflammation as defined by CRP > 5 ng/mL. Geometric mean hepcidin for the overall group was 3.86 ng/mL [95% confidence interval 1.01–16.39]; in iron replete individuals it was 4.44 ng/mL [4.30–4.59] compared with 2.27 ng/mL [2.14–2.40] in iron deficient children (*P* < 0.001) and 2.81 ng/mL [2.62–3.01] in anemic children.

**Table 1:** Summary of patient characteristics and iron indices

Variable		
Age, Mean [2.5, 97.5 centile] (years)		15.9 [14, 19]
Female, *n*/*N* (%)		1166/2273 (51.2)
Hemoglobinopathy		
β‐thalassemia trait, *n*/*N* (%)		118/2263 (5.2)
α‐thalassemia trait, *n*/*N* (%)		474/2198 (21.6)
α‐homozygous or compound heterozygous thalassemia, *n*/*N* (%)		20/2198 (0.9)
HbE, *n*/*N* (%)		28/2273 (1.2)
**Iron parameters**		
**Variable**	**Mean [2.5, 97.5 centile]**	**Abnormal *n*/*N* (%)** b
Hepcidin (ng/mL)	3.86^a^ [1.01, 16.39]	
Ferritin (ng/mL)	24.1^a^ [2.76, 106.57]	585/2210 (26.5)
CRP (mg/L)	0.42^a^ [0.07, 7.12]	74/2117 (3.5)
Hb (g/dL)	13.58 [10.4, 17.1]	439/2217 (19.8)
sTfR (mg/L)	1.99^a^ [1.04, 5.13]	884/2255 (39.4)
sTfR‐F index	1.52^a^ [0.63, 6.29]	563/2203 (25.6)
Iron Deficiency (combined definition),^c^ *n*/*N* (%)		422/2203 (19.2)

a Geometric mean.

b Ferritin < 15 ng/mL; CRP > 5 mg/L; Hb < 12 g/dL in girls and in boys 12–14 years, Hb < 13 g/dL in boys 14–19 years; sTfR > 2.074 mg/L; sTfR‐F > 2.

c Ferritin < 15 ng/mL, or ferritin < 30 ng/mL if CRP > 5 mg/L, and sTfR‐F > 2.

Of our sample, 5.2% had β‐thalassemia trait, 21.6% α^+^ thalassemia trait (heterozygous), 0.9% were α^+^ homozygous or compound heterozygous thalassemia, and 1.2% HbE trait. Hepcidin was suppressed in non‐iron deficient β‐thalassemia trait carriers (4.1 ng/mL) compared with non‐iron deficient children without hemoglobinopathy (5.2 ng/mL, *P* < 0.001) (Supporting Information Table I). This effect was also seen in the non‐iron deficient α‐thalassemia carriers compared to the non‐iron deficient samples without hemoglobinopathy (4.8 ng/mL vs 5.3 ng/mL, *P* = 0.02) (Supporting Information Figure 1).

### 3.1 Associations with hepcidin

We evaluated associations of hepcidin with demographic characteristics and erythropoietic, iron, and inflammatory indices (Table 2). By univariate linear regression, hepcidin was associated with age, sex, Hb, ferritin, CRP, sTfR, and sTfR‐F index. By stepwise multiple regression analysis, hepcidin was associated with ferritin, sTfR, Hb, and CRP with an adjusted *R* ^2^ = 0.259 (Table 2). Hepcidin was not associated with age, sex or carriage of hemoglobinopathy after adjustment, indicating that these factors mediate their effect on hepcidin via iron stores and erythropoiesis.

**Table 2:** Associations between hepcidin and participant characteristics and iron indices

Variable	Regression Coefficient	95% CI	*P*	*β* coefficient^a^
Univariate linear regression for log_10_(hepcidin)				
Age (years)	0.048	0.025, 0.070	<0.001	0.090
Sex^b^	−0.24	−0.30, −0.18	<0.001	−0.16
Hb (g/dL)	0.13	0.11, 0.15	<0.001	0.30
log_10_(ferritin)	0.37	0.34, 0.40	<0.001	0.46
log_10_(sTfR)	−0.50	−0.58, −0.43	<0.001	−0.27
log_10_(sTfR‐F)	−0.52	−0.56, −0.47	<0.001	−0.43
log_10_(CRP)	0.15	0.12, 0.18	<0.001	0.23
β‐thalassemia trait	−0.02	−0.16, 0.12	0.76	−0.006
HbE trait	0.15	−0.12, 0.43	0.27	0.023
α‐thalassemia trait	0.12	0.044, 0.19	0.002	0.066
α‐homozygous or compound heterozygous thalassemia	0.49	0.16, 0.81	0.003	0.063
Multiple linear regression model for log_10_(hepcidin)				
Log_10_(ferritin)	0.27	0.24, 0.30	<0.001	0.34
Log_10_(sTfR)	−0.25	−0.32, −0.17	<0.001	−0.13
Hb (g/dL)	0.05	0.04, 0.07	<0.001	0.13
Log_10_(CRP)	0.10	0.07, 0.12	<0.001	0.15

All variables were included in the multiple regression analysis. Only variables with *P* < 0.05 were included in the final fitted model.

a Variables standardized to have a variance of 1, allowing comparison of regression coefficients between variables.

b Coded variable: Male = 0; Female = 1.

### 3.2 Hepcidin as a diagnostic test of iron deficiency

**Figure 2:**
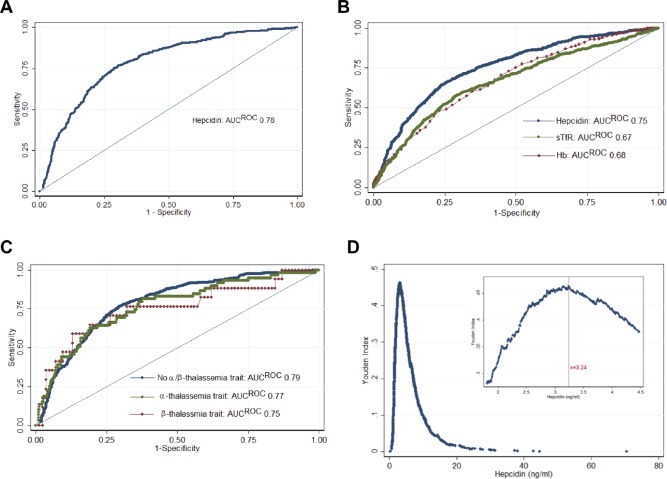
ROC curves for hepcidin to identify iron deficiency. A, Hepcidin compared to ferritin<15 ng/mL, or <30 ng/mL if CRP>5 mg/L, and sTfR‐F index>2 (AUC^ROC^ 0.78) (*n* = 2203) B, using ferritin<15 ng/mL as the gold standard, comparing hepcidin (AUC^ROC^ 0.75) to sTfR (AUC^ROC^ 0.66, *P* < 0.001) and Hb (AUC^ROC^ 0.68, *P* < 0.001) (*n* = 2149) C, ROC curve comparing the performance of hepcidin at distinguishing iron deficiency in samples without α‐ or β‐thalassemia trait (AUC^ROC^ 0.79, *n* = 1620) with samples with α‐ (AUC^ROC^ 0.77, *n* = 468) and with β‐thalassemia trait (AUC^ROC^ 0.75, *n* = 101) (not significant between any group). D, Youden indices [(sensitivity + specificity) − 1] at each cutoff of hepcidin. (Inset) Youden indices in the range of 1.8–4.5 ng/mL. The maximal Youden index was 0.463 and occurred at a hepcidin cutoff of 3.24 ng/mL (sensitivity, 76.8%; specificity, 69.6%). ROC = Receiver Operating Characteristic; AUC = Area Under Curve. [Color figure can be viewed at wileyonlinelibrary.com]

We generated ROC curves and measured the AUC^ROC^ for hepcidin as a test for iron deficiency with the combined definition (Figure 2A). The AUC^ROC^ was 0.78 [0.76–0.81]. We next compared the performance of hepcidin as a diagnostic test of iron deficiency to other indices of iron status (defined by the “ferritin alone”). Hepcidin (AUC^ROC^ 0.75 [0.73–0.77]) performed significantly better than either Hb (AUC^ROC^ 0.68 [0.65–0.70], *P* < 0.001) or sTfR (AUC^ROC^ 0.66 [0.64–0.69], *P* < 0.001) (Figure 2B). The diagnostic test accuracy of hepcidin was not different when AUC^ROC^ were compared by sex, or anemia or carriage of ß‐thalassemia, α‐thalassemia, and HbE (Figure 2C) (Supporting Information Table II).

We next estimated the diagnostic properties of several potential hepcidin thresholds. Hepcidin < 3.2 ng/mL achieved the maximum Youden index (0.46) (Figure 2D), and correctly classified 71.3% of samples, with a sensitivity of 75.6% and a specificity of 70.2%. Alternative thresholds could be selected which yielded different sensitivity and specificity (Supporting Information Table III). At the same hepcidin cutoff, 72.1% of the β‐thalassemia carriers were correctly classified, with a sensitivity of 76.2% and specificity of 71.3%.

To compare this threshold to previous data, we analyzed the hepcidin concentrations of twenty‐eight samples using both the DRG and Bachem assays (Supporting Information Figure 2). Although absolute hepcidin values differ between the two assays, there was a linear correlation (*R* ^2^ = 0.92) between results returned by the two assays. We previously identified an optimal hepcidin threshold, using the Bachem assay, of 5.5 ng/mL (based on the Youden index) in African pre‐school children.^13^ This threshold corresponds to a hepcidin value of 3.1 ng/mL with the DRG assay, which is similar to the cutoff identified in the Sri Lankan population.

Finally, we sought to establish the clinical implications if iron treatment was stratified in this adolescent population using hepcidin, compared with universal distribution of iron or distribution predicated on detection of anemia (Supporting Information Table IV). Provision of iron only to individuals with hepcidin < 3.2 ng/mL prevented about 60% of total individuals from receiving iron whilst ensuring at least three quarters of iron deficient individuals received iron. Had screening been undertaken using testing for anemia, only 36% of iron deficient individuals would receive iron. Importantly, if Hb screening alone had been undertaken, directing iron on the basis of anemia would result in two thirds of iron‐replete ß‐thalassemia carriers (in whom iron supplementation may result in loading) receiving iron, whereas directing iron based on hepcidin could reduce this to only 29% of carriers. Likewise, hepcidin‐directed iron interventions would reach 68% of alpha thalassemia carriers (compared with 40% based on anemia‐screening), although the number of iron replete alpha thalassemia carriers receiving iron would be increased (from 13% to 28%).

## 4 Discussion

In a large cross‐sectional study of adolescents across Sri Lanka, we assessed regulation of serum hepcidin concentrations and the value of this biomarker as an index of iron deficiency. At the population level, associations with serum hepcidin concentration represent known regulation of its gene expression by iron, erythropoiesis, and inflammation. Performance of hepcidin as an index of iron deficiency in this population was not perturbed by carriage of thalassemia. Importantly, the threshold for hepcidin we identified was similar to previously reported cutoffs identified in studies in Africa and elsewhere in Asia. This is the largest diagnostic test accuracy study for hepcidin as a test of iron deficiency, the first such study in South Asia, and the first in a setting where hemoglobinopathy is endemic.

Our data confirm at the population level the known mechanistic regulation of hepcidin, which thereby controls iron absorption and recycling in a manner that represents the net output of multiple integrated signals.^19^ Population level associations between hepcidin and iron stores and inflammation have likewise been demonstrated in developed contexts.^20^ As the direct mediator of iron absorption and recycling, hepcidin provides direct insight into iron physiology, particularly utilization of oral iron. Hepcidin can be used to predict iron uptake and utilization,^21^, ^22^ as well as to distinguish iron deficient children within a population with a high burden of anemia and mixed iron deficiency and inflammation/infection.^13^

Several studies have previously evaluated the performance of hepcidin as an index of iron deficiency, in both population and clinical contexts, and interestingly, performance and optimal thresholds for hepcidin to detect iron deficiency appear similar across different populations and study designs, indicating biologic suppression of hepcidin in iron deficiency.^13^, ^23^ Multiple commercial hepcidin assays exist and the absolute measurements they provide correlate well between assays, however the different assay systems are not directly comparable without harmonization.^5^ Choi *et al* (*n* = 59) reported an AUC^ROC^ = 0.85 for hepcidin to detect iron deficiency in Korean children aged 5 months to 17 years; a cutoff of ≤6.895 ng/mL (using the Bachem assay) had a sensitivity of 79.2% and specificity of 82.8%.^23^ In pre‐selected Egyptian children (*n* = 100), urinary hepcidin had an AUC^ROC^ of 0.84, 0.94, and 1.00 to detect mild and moderate ID, and IDA, respectively.^24^ In Australia, non‐anemic female blood donors had an AUC^ROC^ of 0.89 to detect iron deficiency (*n* = 261).^25^ Wolff *et al* studied healthy adult volunteers (*n* = 33) and found that plasma and urine hepcidin had an AUC^ROC^ to detect iron deficiency (defined as ferritin < 30 μg/L) of 0.94 and 0.93, respectively.^26^ Jonker *et al* compared hepcidin with bone marrow iron stores in 87 non‐inflamed Malawian children undergoing elective surgery, and found that ferritin and sTfR outperformed hepcidin as an index of iron deficiency, although hepcidin was still a potentially useful biomarker. Utilizing similar gold standard definitions of iron deficiency, we studied 1313 Gambian and Tanzanian pre‐school children, and observed that the AUC^ROC^ for hepcidin to identify iron deficiency was 0.85, with a threshold using the Bachem ELISA of 5.5 ng/mL to distinguish iron deficiency across the overall population and among anemic children.^8^, ^23^ Current work is in progress to harmonize hepcidin measurements across the range of different kits and platforms presently available.^16^ A threshold commutable to the value identified in this study is therefore likely to be an appropriate candidate for diagnosis of iron deficiency.

Compared with studies in Africa, we found a lower AUC^ROC^ for hepcidin to diagnose iron deficiency in this study. Unlike the African population, anemia, and inflammation were uncommon in this study; as such, distinction in hepcidin between cases with iron deficiency (which produces hepcidin suppression) and cases with inflammation and iron loading (with elevations in hepcidin) may have been less discrete. The prevalence of iron deficiency based on our definition was relatively low in this study; this is reflected here by low positive predictive value for low hepcidin levels, which is typically the case when evaluating tests in populations where the disease of interest is uncommon. Hepcidin was a more accurate diagnostic test for iron deficiency than hemoglobin, as we also observed in Africa. This reflects an emerging realization that in the public health, anemia, and iron deficiency are not synonymous, and often only a minority of cases of anemia are attributable to iron deficiency^1^; the corollary is that many cases of iron deficiency exist without overt anemia. These data indicate that hemoglobin concentration should not be used alone to identify iron deficiency.

Carriers of thalassemia have mildly increased erythropoiesis (reflected by elevations in sTfR without low ferritin) due to modest ineffective erythropoiesis with an imbalance of globin chains. Elevations in erythropoiesis and mild anemia likely cause an increase in the expression of the bone marrow‐derived hormone erythroferrone, which acts to suppress expression of hepcidin.^10^ This represents a milder phenotype to the condition seen in homozygote or compound heterozygote thalassemia conditions.^27^ We were concerned that suppression of hepcidin in patients carrying thalassemia could impair its utility to detect iron deficiency in the same population. This distinction is important as carriers of thalassemia may have increased iron absorption due hepcidin suppression. Reassuringly, our data indicated that the AUC^ROC^ for hepcidin to detect iron deficiency was not different between thalassemia carriers and controls, and the optimal hepcidin threshold was similar in carriers and non‐carriers. Furthermore, unlike hemoglobin, hepcidin measurement can help direct iron away from iron‐replete thalassemia carriers for whom iron supplementation may be harmful.

Guided by the Youden index, a hepcidin cutoff of 3.2 ng/mL had the highest simultaneous specificity and sensitivity. However, this threshold represents a tradeoff between sensitivity and specificity and selection of the appropriate cutoff ultimately depends on the clinical or public health scenario to which it will be applied, based on the risk of misclassifying an iron replete individual as iron deficient or vice versa. In this study, our reference (gold standard) was a combination of ferritin and soluble transferrin receptor. These assays reflect bone marrow iron stores and tissue iron need. Although thresholds used to define iron deficiency with these indices remain unclear and subject to ongoing re‐consideration,^28^ it would not have been feasible to measure bone marrow iron in this large cross‐sectional population study. The definitions of iron deficiency we used here are widely accepted and have been previously deployed in studies evaluating hepcidin as a diagnostic test.

In Sri Lankan adolescent children, the prevalence of anemia was below the threshold for which a routine universal iron intervention programme (for example daily or weekly iron supplementation) would be considered. Indeed, because this study was enriched with patients likely to carry hemoglobinopathies, the population prevalence of anemia and iron deficiency in Sri Lanka is even lower still. Importantly, as anemia is a poor index of iron status, it is also a poor guide for appropriate allocation of iron interventions in this setting. Strategies for optimally directing iron interventions should ensure iron is preferentially distributed to individuals that need it but not to those who are iron replete (or infected) and who would not benefit, or indeed in whom it might be harmful. For this reason, we are currently prospectively evaluating this approach in Gambian children^29^ and pregnant women.^30^ These trials together with the present diagnostic test accuracy study may help develop the role of hepcidin to classify individuals “ready to receive iron” for optimal stratification in iron distribution programs.

## Author contributors

DW, HD, SP, AMP, and AP designed the research.

AP, LP, RR, GG, LR conducted research (field).

KW, AA, AEA, CF, EE, and CW conducted research (laboratory analysis).

KW, SP, AA, DW, and HD analyzed data.

KW, SP, HD wrote paper and had primary responsibility for final content.

All authors contributed to interpretation of the data and approved the final manuscript.

## Conflict of interests

All authors declare that they have no competing interests.

## Supporting information

Supporting Information
