# Supplementary material for: Hepcidin detects iron deficiency in Sri Lankan adolescents with a high burden of hemoglobinopathy: A diagnostic test accuracy study
Source: Am J Hematol. 2017 Jan 17;92(2):196–203. doi: 10.1002/ajh.24617 (PMC5324588; doi:10.1002/ajh.24617)
Supplement: Supplementary file 1 — Supporting Information [file AJH-92-196-s001.docx]

**
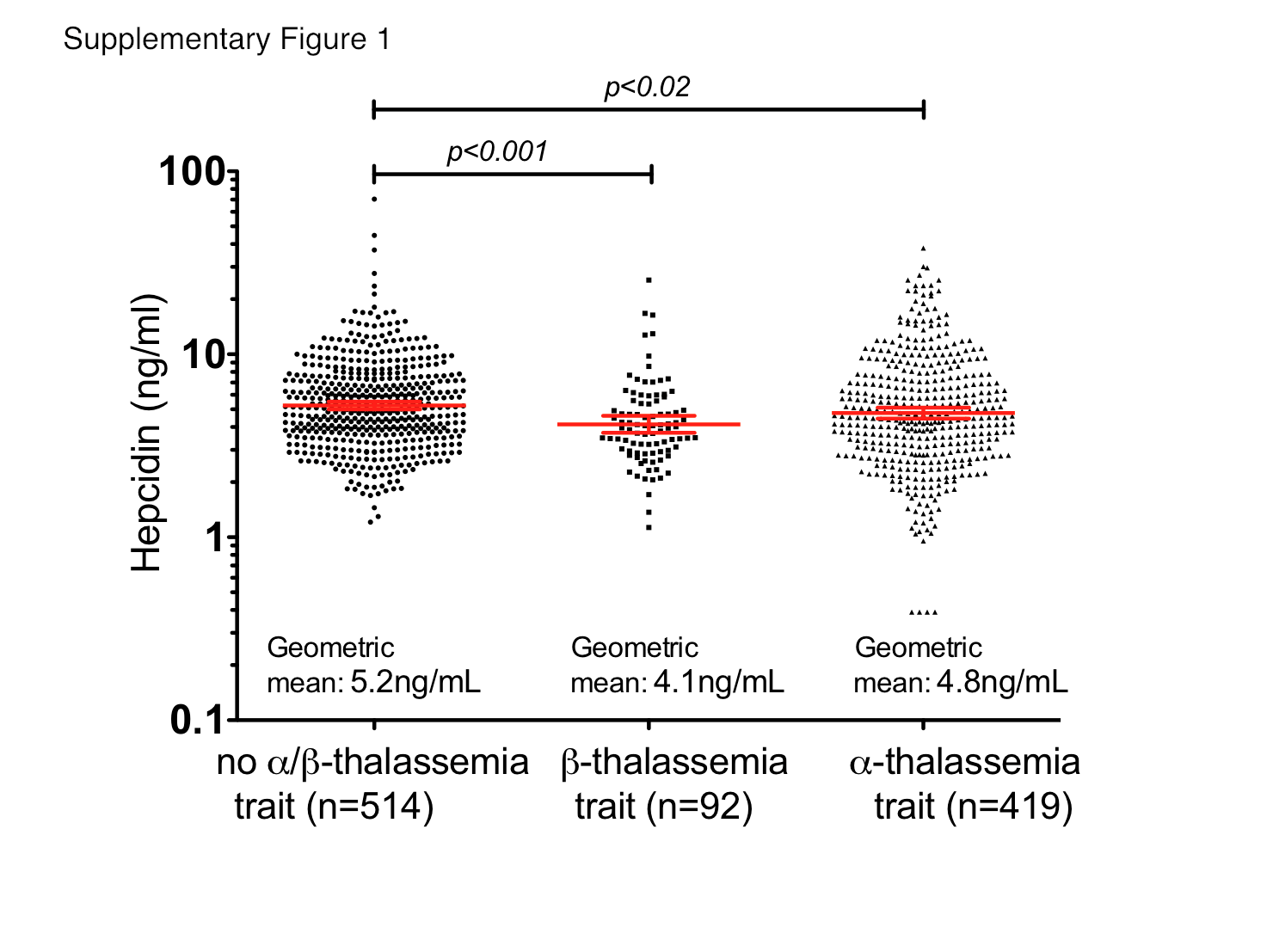
**

**
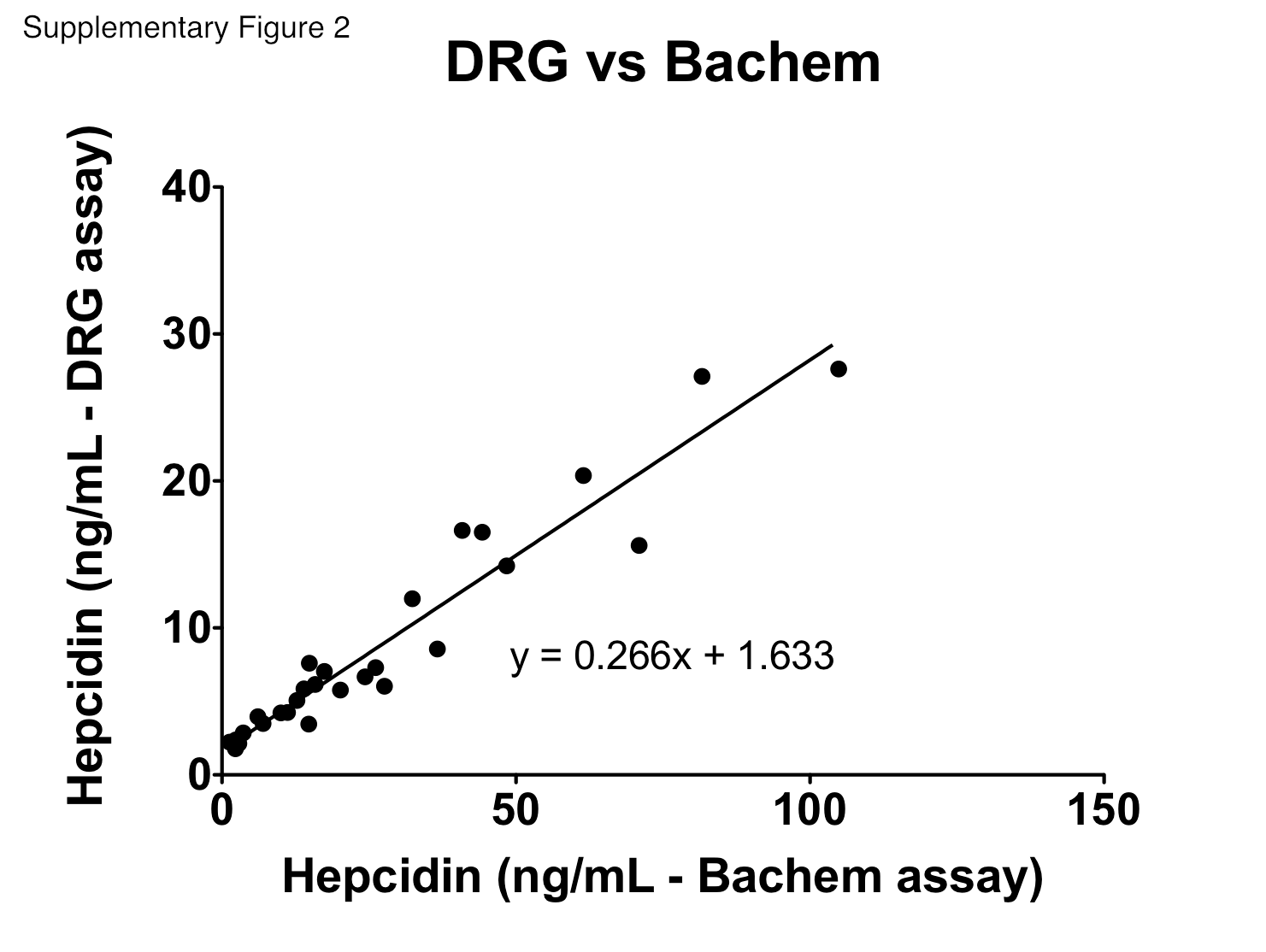
**

**Supplementary Table I. Iron indices in non-iron deficient^2^ samples with and without β-thalassaemia trait, and with and without α-thalassaemia trait.**

| Variable | β-thalassaemia | | | | | α-thalassaemia | | | | |
| --- | --- | --- | --- | --- | --- | --- | --- | --- | --- | --- |
|  | **Without**  **n=514** | **2.5, 97.5 centile** | **With**  **n=92** | **2.5, 97.5 centile** | **P** | **Without**  **n=478** | **2.5, 97.5 centile** | **With**  **n=419** | **2.5, 97.5 centile** | **P** |
| Hepcidin^1^ | 5.2 | 1.9, 16.1 | 4.1 | 1.5, 16.6 | <0.001 | 5.3 | 1.9, 15.3 | 4.8 | 1.1, 22.3 | 0.02 |
| Ferritin^1^ | 36.9 | 10.4, 144.7 | 37.4 | 13.3, 168.0 | 0.86 | 36.7 | 10.4, 144.5 | 33.2 | 10.3, 105.6 | 0.02 |
| sTfR^1^ | 1.8 | 1.0, 5.3 | 2.6 | 1.4, 5.7 | <0.001 | 1.8 | 1.0, 5.1 | 1.7 | 1.1, 3.3 | 0.008 |
| sTfR-F^1^ | 1.2 | 0.6, 3.0 | 1.7 | 0.8, 3.8 | <0.001 | 1.2 | 0.6, 3.0 | 1.2 | 0.6, 2.2 | 0.4 |
| Hb (g/dL) | 14.7 | 12.2, 18.0 | 11.9 | 9.9, 16.6 | <0.001 | 14.7 | 12.2, 18.0 | 13.8 | 11.2, 16.8 | <0.001 |
| Hepcidin:ferritin^1^ | 0.14 | 0.05, 0.6 | 0.11 | 0.03, 0.4 | 0.0011 | 0.14 | 0.05, 0.6 | 0.14 | 0.3, 0.7 | 0.97 |

^1^ Geometric mean

^2^ ID defined as ferritin<15ng/mL, or <30ng/mL if CRP>5mg/l, and sTfR-F>2

**Supplementary Table II. Area under ROC curves comparing performance of hepcidin as an index of iron deficiency in children of different sex, anemia or hemoglobinopathy status**

| Condition | Observations | AUC^ROC^ | 95% CI | P |
| --- | --- | --- | --- | --- |
| No β-thalassemia trait | 2078 | 0.79 | 0.76, 0.81 | 0.90 |
| β-thalassemia trait | 115 | 0.78 | 0.65, 0.90 |  |
| No α-thalassemia^1^ | 1648 | 0.78 | 0.76, 0.81 | 0.78 |
| α-thalassemia | 482 | 0.77 | 0.71, 0.84 |  |
| No hemoglobinopathy^2^ | 1543 | 0.78 | 0.76, 0.81 | 0.58 |
| All hemoglobinopathy | 583 | 0.77 | 0.71, 0.82 |  |
| Male | 1075 | 0.76 | 0.72, 0.81 | 0.77 |
| Female | 1114 | 0.77 | 0.74, 0.80 |  |
| Non-anemic^3^ | 1732 | 0.77 | 0.74, 0.80 | 0.50 |
| Anemic | 415 | 0.75 | 0.71, 0.80 |  |

^1^ 3.7 heterozygous (n=416), 4.2 heterozygous (n=46), 3.7 homozygous (n=14), compound heterozygous (n=6)

^2^ β-thalassemia trait (n=115), α-thalassemia (n as above^1^), HbE trait (n=26), S thalassemia trait (n=5)

^3^ Hb<12g/dL in girls & in boys 12-14 yrs, Hb<13g/dL in boys 14-19 yrs

**Supplementary Table III. Properties of different cutoffs of hepcidin as a diagnostic test of iron deficiency in this population**^1^

| Cutoff (ng/mL) | Sensitivity | 95% CI | Specificity | 95% CI | Positive predictive value | 95% CI | Negative predictive value | 95% CI | Positive likelihood ratio (%) | Negative likelihood ratio (%) | Correctly classified (%) | Youden index |
| --- | --- | --- | --- | --- | --- | --- | --- | --- | --- | --- | --- | --- |
| <2.0 | 43.4 | 38.6, 48.2 | 88.7 | 87.2, 90.1 | 47.7 | 42.6, 52.8 | 86.9 | 85.2, 88.4 | 3.84 | 0.64 | 80.0 | 0.320 |
| <2.5 | 60.0 | 55.1, 64.7 | 81.4 | 79.5, 83.1 | 43.2 | 39.2, 47.4 | 89.6 | 88.0, 91.0 | 3.22 | 0.49 | 77.3 | 0.413 |
| <3.0 | 72.3 | 67.7, 76.5 | 72.9 | 70.8, 75.0 | 38.8 | 35.3, 42.3 | 91.7 | 90.2, 93.1 | 2.67 | 0.38 | 72.8 | 0.452 |
| <3.1^2^ | 74.2 | 69.7, 78.3 | 71.5 | 69.4, 73.6 | 38.2 | 34.8, 41.6 | 92.1 | 90.6, 93.5 | 2.61 | 0.36 | 72.0 | 0.458 |
| <3.2 | 75.6 | 71.2, 79.6 | 70.2 | 68.1, 72.4 | 37.6 | 34.3, 40.9 | 92.4 | 90.8, 93.7 | 2.54 | 0.35 | 71.3 | 0.459 |
| <3.3 | 77.0 | 72.7, 80.9 | 68.6 | 66.3, 70.7 | 36.7 | 33.5, 40.0 | 92.6 | 91.1, 94.0 | 2.45 | 0.34 | 70.2 | 0.453 |
| <3.5 | 79.1 | 75.0, 82.9 | 65.4 | 63.2, 67.6 | 35.2 | 32.1, 38.3 | 93.0 | 91.4, 94.3 | 2.29 | 0.32 | 68.0 | 0.446 |
| <4.0 | 84.6 | 80.8, 87.9 | 57.6 | 55.2, 59.9 | 32.1 | 29.3 34.9 | 94.0 | 92.5, 95.4 | 1.99 | 0.27 | 62.7 | 0.421 |
| <4.5 | 87.9 | 84.4, 90.9 | 49.9 | 47.5, 52.2 | 29.4 | 26.9, 31.9 | 94.6 | 92.9, 95.9 | 1.75 | 0.24 | 57.1 | 0.378 |

^1^ Iron deficiency defined as ferritin<15ng/mL, or <30ng/mL if CRP>5mg/L, and sTfR-F>2

^2^ A hepcidin concentration of 3.1ng/mL reported by the DRG hepcidin 25 (bioactive) ELISA corresponds to a hepcidin concentration of 5.5ng/mL measured using the Bachem hepcidin-25 (human) EIA kit (see Supplemental Figure 2)

Sensitivity (correctly identified positives), Specificity (correctly identified negatives), Positive predictive value (proportion of true positives), Negative predictive value (proportion of true negatives), Positive likelihood ratio (sensitivity / (specificity – 1), Negative likelihood ratio ((1-sensitivity) / specificity) and Youden index (sensitivity + specificity – 1) are shown. The maximum Youden index was 0.463 at a hepcidin cutoff of 3.24ng/mL.

**Supplementary Table IV. Proportions of study population subgroups receiving iron based on policies of iron supplementation to all children, to anemic children or to children with low hepcidin levels**

| **Subgroup of study population** | **Universal iron distribution** | **Iron administered if anemic^1^** | **Iron administered if**  **hepcidin<3.2ng/mL** |
| --- | --- | --- | --- |
| Overall population | 100% | 439/2217 (19.8%) | 899/2273 (39.6%) |
| Iron deficient^2^ | 100% | 148/413 (35.8%) | 319/422 (75.6%) |
| Non-iron deficient^2^ | 100% | 267/1734 (15.4%) | 530/1781 (29.8%) |
| ß-thalassemia trait | 100% | 74/105 (70.5%) | 45/118 (38.1%) |
| α-thalassemia trait | 100% | 87/492 (17.7%) | 170/494 (34.4%) |
| ß-thalassemia trait - iron replete | 100% | 55/82 (67.0%) | 27/94 (28.7%) |
| α-thalassemia trait - iron replete | 100% | 56/417 (13.4%) | 119/419 (28.4%) |
| ß-thalassemia trait - Iron deficient^2^ | 100% | 16/20 (80.0%) | 16/21 (76.2%) |
| α-thalassemia trait - Iron deficient^2^ | 100% | 25/63 (40.0%) | 43/63 (68.3%) |

^1^Hb<12g/dL in girls & in boys 12-14 yrs, Hb<13g/dL in boys 14-19 yrs

^2^Ferritin<15ng/mL, or ferritin<30ng/mL if CRP>5mg/L, & sTfr
